# Supplementary material for: Using recurrent neural networks to detect supernumerary chromosomes in fungal strains causing blast diseases
Source: NAR Genom Bioinform. 2024 Aug 20;6(3):lqae108. doi: 10.1093/nargab/lqae108 (PMC11333962; doi:10.1093/nargab/lqae108)
Supplement: lqae108_Supplemental_Files [file lqae108_supplemental_files.zip › Suppl_figures_rev2.2.docx]

Using recurrent neural networks to detect supernumerary chromosomes in fungal strains causing blast diseases

Gyawali et al.

**Figure S1. Electrophoretic profile of TF05-1**

Pulsed-field electrophoresis was employed using the CHEF Mapper XA system (Bio-Rad, cat no. 1703670) with parameters (7.5 °C, 1.5 V, 120°, Int SW 1200 s, Fin SW 4800, total time 192 hours) to achieve a separation of mini-chromosomes. The CHEF DNA size standards, H. wingei Ladder (Bio-Rad, Cat no. 1703667), ranging from 1 to 3.1 Mb was used to estimate the size of chromosomes. The red arrow indicates the presence of a chromosome of approximately 1.3 Mb. The genome assembly of TF05-1 revealed three contigs (mini2a, mini2b, mini2c) presumably from a second mini-chromosome. Copy number analysis indicates that mini2b was duplicated in the TF05-1 genome. Therefore, the size of the second mini-chromosome was estimated to be larger than 3.13 Mb, which could not be separated from core-chromosomes in the current CHEF running conditions.


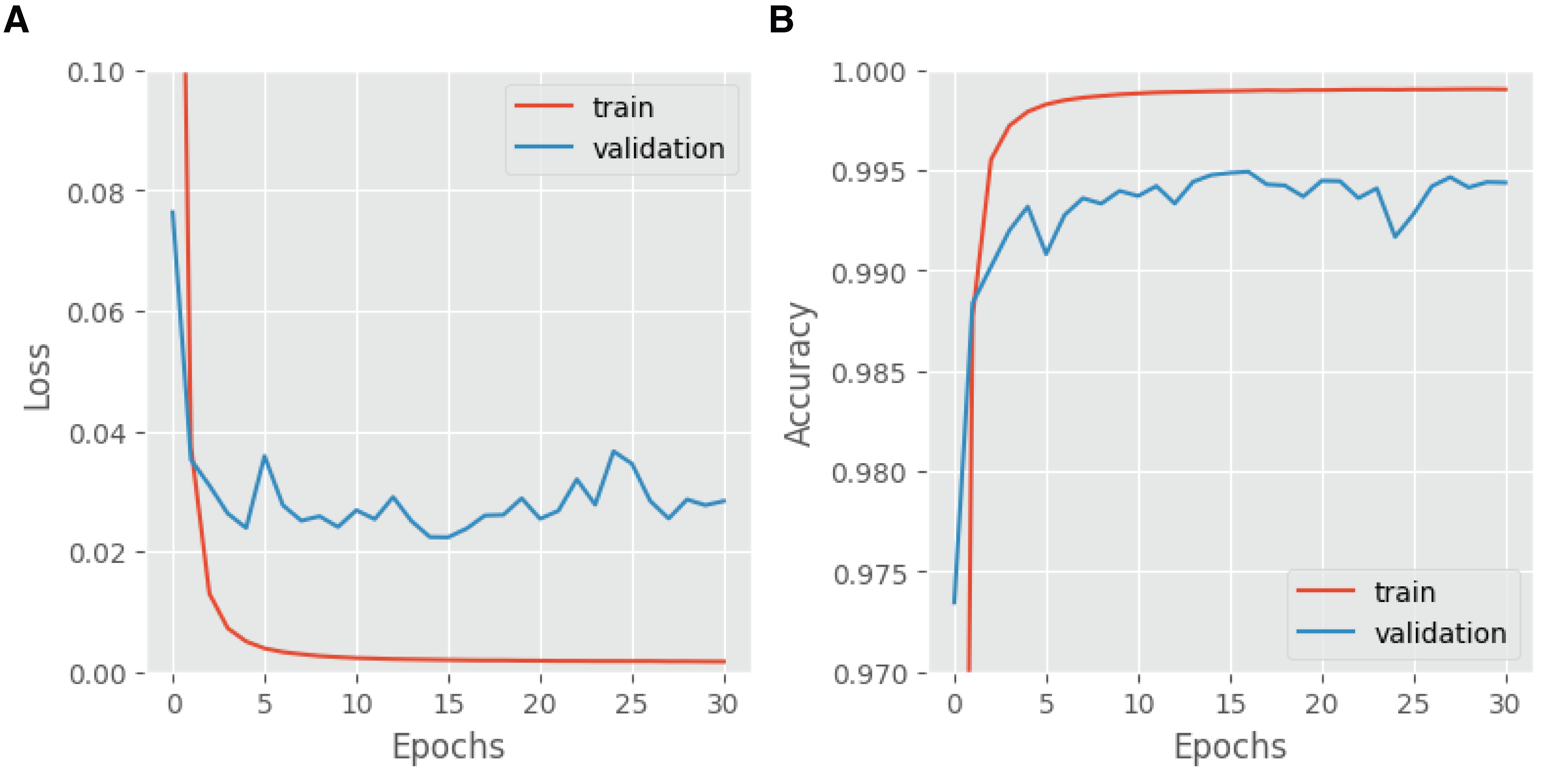


**Figure S2. Prediction loss and accuracy of models during training**

Plot of (**A**) Loss and (**B**) Accuracy at the end of each epoch during the training of the model with 99 bp sequences each of which consists of eleven 9-mer tokens. As shown in (A), the model's performance did not improve after epoch 15 until epoch 30 and the model training stops after 30 epochs. The checkpoint at epoch 15 is restored as the best model.

**Figure S3.** **MiniC proportions and recall rates with different probability thresholds**

By default, the probability of 0.5 was used for the classification. The probability to classify a sequence to be from a mini-chromosome was increased. Plot (**A**) shows the changes of miniC proportions of multiple strains over different probability thresholds; plot (**B**) shows the changes of adjusted recall rates, each of which was the ratio of the number of sequences classified as mini-chromosome sequences using a probability threshold indicated in X-axis to that using the probability threshold of 0.5. Strains indicated in blue had no mini-chromosomes, while strains indicated in purple had at least one mini-chromosome. The recall rates drop faster when the probability threshold increase. Therefore, the probability of 0.99 was finally selected as the threshold for the classification.
